# Diethyl 1,8-bis­(4-methyl­phen­yl)-11-oxatricyclo­[6.2.1.0^2,7^]undeca-2,4,6-triene-9,10-dicarboxyl­ate

**DOI:** 10.1107/S1600536813005291

**Published:** 2013-03-06

**Authors:** B. Balakrishnan, Meganathan Nandakumar, Pandamangalam R. Seshadri, Arasambattu K. Mohanakrishnan

**Affiliations:** a Department of Physics, P.T. Lee Chengalvaraya Naicker College of Engineering and Technology, Kancheepuram 631 502, India; b Department of Organic Chemistry, University of Madras, Guindy Campus, Chennai 600 025, India; c Postgraduate and Research Department of Physics, Agurchand Manmull Jain College, Chennai 600 114, India

## Abstract

The title compound, C_30_H_30_O_5_, is the Diels–Alder adduct from 1,3-diphenyl­benzo[*c*]furan and diethyl maleate. The mol­ecule comprises a fused tricyclic system containing two five-membered rings, which are in envelope conformations with the O atom at the flap, and a six-membered ring adopting a boat conformation. The dihedral angle between the 4-methyl­phenyl substituents in the 1- and 8-positions is 62.1 (1)°. The ethyl group of one ester group and the eth­oxy group of the other ester group are disordered over two sets of sites, with occupancy ratios of 0.43 (2):0.57 (2) and 0.804 (7):0.196 (7), respectively. In the crystal, inversion dimers are formed through pairs of C—H⋯O inter­actions.

## Related literature

For background to Diels–Alder reactions, see: Akio & Toshiki (2010 ▶). For related structures, see: Bailey *et al.* (1995 ▶); Takahashi *et al.* (2003 ▶); Simpson *et al.* (2004 ▶); Toze *et al.* (2010 ▶). For puckering and asymmetry parameters, see: Cremer & Pople (1975 ▶); Nardelli (1983 ▶).
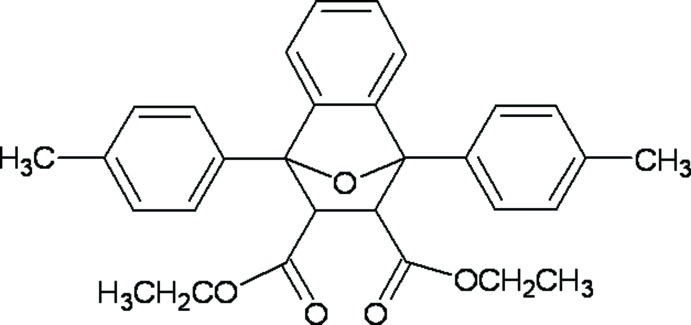


## Experimental

### 

#### Crystal data

C_30_H_30_O_5_
*M* *_r_* = 470.54
Triclinic, 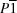

*a* = 9.8722 (3) Å
*b* = 10.7413 (3) Å
*c* = 13.3081 (3) Å
α = 109.319 (1)°
β = 105.045 (1)°
γ = 90.374 (1)°
*V* = 1279.45 (6) Å^3^
*Z* = 2
Mo *K*α radiation
μ = 0.08 mm^−1^
*T* = 293 K
0.30 × 0.20 × 0.20 mm

#### Data collection

Bruker Kappa APEXII CCD diffractometer
Absorption correction: multi-scan (*SADABS*; Bruker, 2004 ▶) *T*_min_ = 0.951, *T*_max_ = 0.953
21475 measured reflections
4505 independent reflections
3754 reflections with *I* > 2σ(*I*)
*R*_int_ = 0.027

#### Refinement

*R*[*F* ^2^ > 2σ(*F* ^2^)] = 0.038
*wR*(*F* ^2^) = 0.112
*S* = 1.03
4505 reflections
360 parameters
84 restraints
H-atom parameters constrained
Δρ_max_ = 0.23 e Å^−3^
Δρ_min_ = −0.18 e Å^−3^

### 

Data collection: *APEX2* (Bruker, 2004 ▶); cell refinement: *SAINT* (Bruker, 2004 ▶); data reduction: *SAINT*; program(s) used to solve structure: *SHELXS97* (Sheldrick, 2008 ▶); program(s) used to refine structure: *SHELXL97* (Sheldrick, 2008 ▶); molecular graphics: *ORTEP-3 for Windows* (Farrugia, 2012 ▶) and *PLATON* (Spek, 2009 ▶); software used to prepare material for publication: *SHELXL97*, *PLATON* and *publCIF* (Westrip, 2010 ▶).

## Supplementary Material

Crystal structure: contains datablock(s) I, global. DOI: 10.1107/S1600536813005291/im2419sup1.cif

Structure factors: contains datablock(s) I. DOI: 10.1107/S1600536813005291/im2419Isup2.hkl

Supplementary material file. DOI: 10.1107/S1600536813005291/im2419Isup3.cml

Additional supplementary materials:  crystallographic information; 3D view; checkCIF report

## Figures and Tables

**Table 1:** Hydrogen-bond geometry (Å, °)

*D*—H⋯*A*	*D*—H	H⋯*A*	*D*⋯*A*	*D*—H⋯*A*
C5—H5⋯O4^i^	0.93	2.66	3.558 (2)	164
C11—H11⋯O2^ii^	0.93	2.66	3.433 (2)	141

Symmetry codes: (i) 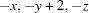; (ii) 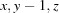.

**Fig. 1.**
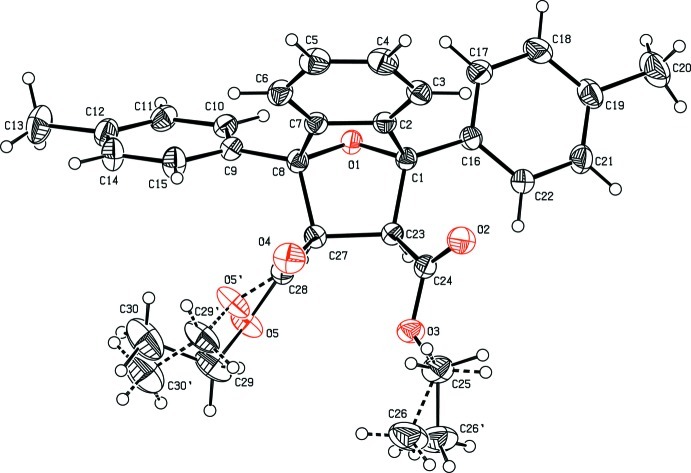
Molecular structure of the title compound, showing 30% probability displacement ellipsoids.

## supplementary crystallographic information

### Comment

Diels-Alder adducts are valuable synthetic intermediates and their use for the synthesis of natural products and of polycyclic aromatic hydrocarbons is well documented (Akio & Toshiki, 2010). The title compound, C_30_H_30_O_5_, comprises a fused tricyclic system and two 4-methylphenyl rings attached to this system (Fig.1). The tricyclic system consists of two 5-membered rings and one aromatic ring. In addition, two ethyl carboxylate units are attached to the tricyclic system. Geometrical parameters agree well with reported structures (Bailey *et al.* 1995; Takahashi *et al.* 2003; Simpson *et al.*, 2004; Toze *et al.*, 2010). The 5-membered ring C_1_\C_2_\C_7_\C_8_\O_1_ adopts an envelope conformation with atom O_1_ displaced by -(0.757) Å from the mean plane of the other ring atoms C_1_\C_2_\C_7_\C_8_. The puckering parameters (Cremer & Pople, 1975) and asymmetry parameters (Nardelli, 1983) are q_2_ = 0.515 (1) Å, φ = 143.2 (2)°, Δ_S_(O_1_) = 0.007 (1)° and Δ_2_(O_1_) = 0.312 (1)°.The second 5-membered ring C_1_\ C_23_\C_27_\C_8_\O_1_ also adopts an envelope conformation with O_1_ displaced by -(0.835) Å from the mean plane of the other ring atoms C_1_\ C_23_\C_27_\C_8_. The puckering parameters (Cremer & Pople, 1975) and asymmetry parameters (Nardelli, 1983) are q_2_ = 0.593 (1) Å, φ = -37.5 (1)°, Δ_S_(O_1_) = 0.012 (1)° and Δ_2_(O_1_) = 0.355 (1)°. The six membered ring C_1_/C_2_/C_7_/C_8_/C_27_/C_23_ adopts a boat conformation with puckering parameter q_2_ = 0.951 (1) Å, θ=89.6 (9)° and φ = 359.9 (9)°.

The dihedral angle between the rings C_1_/C_2_/C_7_/C_8_/O_1_ and C_1_/C_23_/C_27_/C_8_/O_1_ is 66.1 (1)°. The dihedral angle between the terminal 4-methylphenyl rings is 62.1 (1)°. One of the aromatic substituents (C9 - C15) is almost orthogonal to the plane formed by the six atoms C1, C2, C7, C8, C27 and C23 of the tricyclic ring, the dihedral angle being 84.8 (1)° (Nardelli, 1983). The atoms O5, C29 and C30 and C26 of ester groups are disordered over two sites with occupancy ratios of 0.804 (7): 0.196 (7) and 0.43 (2): 0.57 (2). The ester group is twisted from the mean plane of the tricyclic ring, with C30 towards C28 as evidenced by the torsion angle C30—C29—O5—C28 = 89.1 (5)°. The second ester group is co-planer with the attached trycyclic ring as evidenced by the torsion angle C24—O3—C25—C26 = -172 (9)°. Centrosymmetric dimers are formed by C—H···O interactions.

### Experimental

1,3-Di-p-tolylisobenzofuran 4 (1.00 g, 3.36 mmole) was dissolved in toluene (25 ml) and treated with 2 equivalents of dimethyl maleate (1.16 g, 1.15 ml, 6.74 mmole). The reaction mixture was refluxed and the reaction was monitored by TLC. After 8 h, the mixture was cooled to room temperature. The solvent was removed and the residue was purified by column chromatography (Silica gel, 10%, EA/hexane) to give the adduct as a white solid. Yield: 1.41 g (89%) and m.p. 179° C. This adduct was crystallized from CHCl_3_/CH_3_OH (3:1) by the slow evaporation method.

### Refinement

All H atoms were positioned geometrically and allowed to ride on their parent atoms, with (C—H= 0.93–0.96 Å), and *U*_iso_(H) = 1.5 *U*_eq_(C) for methyl H atoms and 1.2U_eq_(C) for other H atoms. In one ester group the ethyl group and in the other ester group the ethoxy group are disordered over two sites with occupancy ratio of 0.43 (2): 0.57 (2), 0.804 (7): 0.196 (7), respectively.

### Crystal data

**Table:**

C_30_H_30_O_5_	*Z* = 2
*M**_r_* = 470.54	*F*(000) = 500
Triclinic, *P*1	*D*_x_ = 1.221 Mg m^−^^3^
Hall symbol: -P 1	Mo *K*α radiation, λ = 0.71073 Å
*a* = 9.8722 (3) Å	Cell parameters from 7905 reflections
*b* = 10.7413 (3) Å	θ = 2.0–25.0°
*c* = 13.3081 (3) Å	µ = 0.08 mm^−^^1^
α = 109.319 (1)°	*T* = 293 K
β = 105.045 (1)°	Block, colourless
γ = 90.374 (1)°	0.30 × 0.20 × 0.20 mm
*V* = 1279.45 (6) Å^3^	

### Data collection

**Table:**

Bruker Kappa APEXII CCD diffractometer	4505 independent reflections
Radiation source: fine-focus sealed tube	3754 reflections with *I* > 2σ(*I*)
Graphite monochromator	*R*_int_ = 0.027
ω and φ scan	θ_max_ = 25.0°, θ_min_ = 2.0°
Absorption correction: multi-scan (*SADABS*; Bruker, 2004)	*h* = −11→11
*T*_min_ = 0.951, *T*_max_ = 0.953	*k* = −12→12
21475 measured reflections	*l* = −15→15

### Refinement

**Table:**

Refinement on *F*^2^	Secondary atom site location: difference Fourier map
Least-squares matrix: full	Hydrogen site location: inferred from neighbouring sites
*R*[*F*^2^ > 2σ(*F*^2^)] = 0.038	H-atom parameters constrained
*wR*(*F*^2^) = 0.112	*w* = 1/[σ^2^(*F*_o_^2^) + (0.0574*P*)^2^ + 0.3047*P*] where *P* = (*F*_o_^2^ + 2*F*_c_^2^)/3
*S* = 1.03	(Δ/σ)_max_ < 0.001
4505 reflections	Δρ_max_ = 0.23 e Å^−^^3^
360 parameters	Δρ_min_ = −0.18 e Å^−^^3^
84 restraints	Extinction correction: *SHELXL97* (Sheldrick, 2008), Fc^*^=kFc[1+0.001xFc^2^λ^3^/sin(2θ)]^-1/4^
Primary atom site location: structure-invariant direct methods	Extinction coefficient: 0.028 (3)

### Special details

**Table:**

Geometry. All e.s.d.'s (except the e.s.d. in the dihedral angle between two l.s. planes) are estimated using the full covariance matrix. The cell e.s.d.'s are taken into account individually in the estimation of e.s.d.'s in distances, angles and torsion angles; correlations between e.s.d.'s in cell parameters are only used when they are defined by crystal symmetry. An approximate (isotropic) treatment of cell e.s.d.'s is used for estimating e.s.d.'s involving l.s. planes.
Refinement. Refinement of *F*^2^ against ALL reflections. The weighted *R*-factor *wR* and goodness of fit *S* are based on *F*^2^, conventional *R*-factors *R* are based on *F*, with *F* set to zero for negative *F*^2^. The threshold expression of *F*^2^ > σ(*F*^2^) is used only for calculating *R*-factors(gt) *etc*. and is not relevant to the choice of reflections for refinement. *R*-factors based on *F*^2^ are statistically about twice as large as those based on *F*, and *R*- factors based on ALL data will be even larger.

### Fractional atomic coordinates and isotropic or equivalent isotropic displacement parameters (Å^2^)

**Table:**

	*x*	*y*	*z*	*U*_iso_*/*U*_eq_	Occ. (<1)
C1	0.16153 (14)	1.11798 (13)	0.42413 (11)	0.0342 (3)	
C2	0.18246 (14)	1.12120 (14)	0.31608 (11)	0.0364 (3)	
C3	0.25194 (16)	1.21282 (16)	0.29039 (13)	0.0452 (4)	
H3	0.2958	1.2926	0.3444	0.054*	
C4	0.25420 (18)	1.18202 (19)	0.18119 (15)	0.0555 (4)	
H4	0.3011	1.2418	0.1616	0.067*	
C5	0.18819 (18)	1.06438 (19)	0.10146 (14)	0.0554 (4)	
H5	0.1923	1.0454	0.0289	0.066*	
C6	0.11570 (17)	0.97368 (17)	0.12708 (13)	0.0482 (4)	
H6	0.0697	0.8949	0.0727	0.058*	
C7	0.11386 (14)	1.00369 (14)	0.23586 (11)	0.0376 (3)	
C8	0.05102 (14)	0.93225 (13)	0.29702 (11)	0.0362 (3)	
C9	0.02041 (15)	0.78454 (14)	0.24879 (12)	0.0393 (3)	
C10	0.08168 (15)	0.70361 (14)	0.30645 (12)	0.0404 (3)	
H10	0.1472	0.7408	0.3753	0.049*	
C11	0.04615 (18)	0.56778 (15)	0.26241 (14)	0.0485 (4)	
H11	0.0881	0.5153	0.3027	0.058*	
C12	−0.04982 (19)	0.50845 (15)	0.16043 (14)	0.0524 (4)	
C13	−0.0889 (3)	0.36078 (18)	0.11335 (19)	0.0792 (6)	
H13A	−0.1722	0.3401	0.0520	0.119*	
H13B	−0.1065	0.3306	0.1695	0.119*	
H13C	−0.0128	0.3174	0.0885	0.119*	
C14	−0.1090 (2)	0.58990 (17)	0.10260 (15)	0.0589 (5)	
H14	−0.1727	0.5524	0.0329	0.071*	
C15	−0.07535 (18)	0.72575 (16)	0.14621 (14)	0.0534 (4)	
H15	−0.1177	0.7782	0.1060	0.064*	
C16	0.27490 (14)	1.19021 (14)	0.52804 (11)	0.0366 (3)	
C17	0.41180 (16)	1.15731 (18)	0.53553 (14)	0.0532 (4)	
H17	0.4309	1.0915	0.4764	0.064*	
C18	0.51974 (18)	1.2206 (2)	0.62912 (15)	0.0632 (5)	
H18	0.6105	1.1958	0.6328	0.076*	
C19	0.49591 (18)	1.32047 (19)	0.71785 (14)	0.0566 (4)	
C20	0.6155 (2)	1.3927 (3)	0.81909 (18)	0.0905 (8)	
H20A	0.6696	1.4543	0.8027	0.136*	
H20B	0.6750	1.3298	0.8398	0.136*	
H20C	0.5779	1.4399	0.8791	0.136*	
C21	0.35987 (18)	1.35224 (17)	0.71005 (13)	0.0525 (4)	
H21	0.3411	1.4185	0.7691	0.063*	
C22	0.25038 (16)	1.28856 (15)	0.61707 (12)	0.0434 (4)	
H22	0.1594	1.3121	0.6144	0.052*	
C23	0.00553 (14)	1.13855 (13)	0.42249 (11)	0.0337 (3)	
H23	−0.0045	1.1398	0.4942	0.040*	
C24	−0.04509 (15)	1.26460 (14)	0.40726 (11)	0.0373 (3)	
C25	−0.24375 (19)	1.38633 (18)	0.39529 (18)	0.0635 (5)	
H25A	−0.2326	1.3968	0.3281	0.076*	0.43 (2)
H25B	−0.2002	1.4658	0.4574	0.076*	0.43 (2)
H25C	−0.2742	1.3743	0.3170	0.076*	0.57 (2)
H25D	−0.1768	1.4649	0.4329	0.076*	0.57 (2)
C26	−0.3945 (9)	1.3647 (15)	0.3868 (18)	0.091 (3)	0.43 (2)
H26A	−0.4391	1.2929	0.3197	0.136*	0.43 (2)
H26B	−0.4385	1.4439	0.3859	0.136*	0.43 (2)
H26C	−0.4040	1.3432	0.4493	0.136*	0.43 (2)
C26'	−0.3651 (11)	1.4015 (12)	0.4408 (11)	0.087 (2)	0.57 (2)
H26D	−0.4239	1.3189	0.4107	0.130*	0.57 (2)
H26E	−0.4183	1.4687	0.4214	0.130*	0.57 (2)
H26F	−0.3328	1.4268	0.5200	0.130*	0.57 (2)
C27	−0.07443 (14)	1.00649 (13)	0.33396 (11)	0.0347 (3)	
H27	−0.1136	0.9575	0.3721	0.042*	
C28	−0.19370 (16)	1.02059 (15)	0.24236 (13)	0.0422 (4)	
O1	0.15563 (9)	0.97526 (9)	0.40324 (7)	0.0361 (2)	
O2	0.02359 (12)	1.34954 (11)	0.39779 (10)	0.0543 (3)	
O3	−0.17832 (11)	1.27117 (10)	0.41131 (9)	0.0479 (3)	
O4	−0.18455 (12)	1.07858 (13)	0.18168 (10)	0.0586 (3)	
O5	−0.3145 (3)	0.9610 (3)	0.2455 (3)	0.0586 (7)	0.804 (7)
C29	−0.4441 (3)	0.9681 (3)	0.1669 (3)	0.0782 (10)	0.804 (7)
H29A	−0.4388	1.0503	0.1522	0.094*	0.804 (7)
H29B	−0.5227	0.9671	0.1980	0.094*	0.804 (7)
C30	−0.4678 (5)	0.8522 (5)	0.0606 (3)	0.1017 (14)	0.804 (7)
H30A	−0.3971	0.8603	0.0247	0.152*	0.804 (7)
H30B	−0.5594	0.8512	0.0126	0.152*	0.804 (7)
H30C	−0.4619	0.7712	0.0765	0.152*	0.804 (7)
O5'	−0.2954 (12)	0.9430 (15)	0.2035 (11)	0.064 (2)	0.196 (7)
C29'	−0.4114 (12)	0.9582 (17)	0.1155 (14)	0.077 (3)	0.196 (7)
H29C	−0.3818	0.9476	0.0494	0.093*	0.196 (7)
H29D	−0.4453	1.0448	0.1395	0.093*	0.196 (7)
C30'	−0.5239 (16)	0.8499 (19)	0.0939 (19)	0.104 (5)	0.196 (7)
H30D	−0.4892	0.7652	0.0683	0.156*	0.196 (7)
H30E	−0.6058	0.8556	0.0385	0.156*	0.196 (7)
H30F	−0.5486	0.8599	0.1612	0.156*	0.196 (7)

### Atomic displacement parameters (Å^2^)

**Table:**

	*U*^11^	*U*^22^	*U*^33^	*U*^12^	*U*^13^	*U*^23^
C1	0.0362 (7)	0.0306 (7)	0.0349 (7)	0.0041 (5)	0.0088 (6)	0.0109 (6)
C2	0.0330 (7)	0.0402 (8)	0.0362 (7)	0.0066 (6)	0.0103 (6)	0.0125 (6)
C3	0.0409 (8)	0.0479 (9)	0.0481 (9)	0.0005 (7)	0.0134 (7)	0.0173 (7)
C4	0.0531 (10)	0.0686 (11)	0.0566 (10)	0.0035 (8)	0.0230 (8)	0.0306 (9)
C5	0.0562 (10)	0.0763 (12)	0.0407 (9)	0.0097 (9)	0.0200 (8)	0.0240 (9)
C6	0.0495 (9)	0.0536 (9)	0.0361 (8)	0.0078 (7)	0.0112 (7)	0.0091 (7)
C7	0.0360 (7)	0.0395 (8)	0.0359 (7)	0.0067 (6)	0.0090 (6)	0.0119 (6)
C8	0.0370 (7)	0.0348 (7)	0.0330 (7)	0.0049 (6)	0.0051 (6)	0.0104 (6)
C9	0.0407 (8)	0.0352 (8)	0.0405 (8)	0.0050 (6)	0.0124 (6)	0.0101 (6)
C10	0.0417 (8)	0.0385 (8)	0.0411 (8)	0.0076 (6)	0.0136 (6)	0.0119 (6)
C11	0.0582 (10)	0.0386 (8)	0.0545 (10)	0.0128 (7)	0.0228 (8)	0.0179 (7)
C12	0.0623 (10)	0.0361 (8)	0.0558 (10)	0.0021 (7)	0.0230 (8)	0.0071 (7)
C13	0.1085 (17)	0.0395 (10)	0.0787 (14)	−0.0028 (10)	0.0258 (13)	0.0067 (9)
C14	0.0661 (11)	0.0458 (9)	0.0473 (10)	−0.0056 (8)	0.0021 (8)	0.0040 (8)
C15	0.0610 (10)	0.0415 (9)	0.0464 (9)	0.0022 (7)	0.0000 (8)	0.0121 (7)
C16	0.0367 (7)	0.0369 (7)	0.0354 (7)	0.0023 (6)	0.0078 (6)	0.0132 (6)
C17	0.0410 (9)	0.0607 (10)	0.0460 (9)	0.0083 (7)	0.0083 (7)	0.0062 (8)
C18	0.0366 (9)	0.0819 (13)	0.0579 (11)	0.0053 (8)	0.0048 (8)	0.0136 (10)
C19	0.0463 (9)	0.0692 (11)	0.0438 (9)	−0.0116 (8)	0.0026 (7)	0.0137 (8)
C20	0.0609 (12)	0.123 (2)	0.0564 (12)	−0.0219 (13)	−0.0039 (10)	0.0063 (12)
C21	0.0569 (10)	0.0503 (9)	0.0393 (9)	−0.0051 (8)	0.0113 (7)	0.0030 (7)
C22	0.0411 (8)	0.0431 (8)	0.0417 (8)	0.0023 (6)	0.0109 (6)	0.0095 (7)
C23	0.0352 (7)	0.0345 (7)	0.0316 (7)	0.0046 (6)	0.0087 (6)	0.0119 (6)
C24	0.0393 (8)	0.0354 (7)	0.0349 (7)	0.0052 (6)	0.0090 (6)	0.0102 (6)
C25	0.0553 (10)	0.0506 (10)	0.0903 (14)	0.0237 (8)	0.0199 (10)	0.0313 (10)
C26	0.051 (4)	0.080 (6)	0.151 (9)	0.023 (3)	0.028 (5)	0.053 (6)
C26'	0.068 (4)	0.083 (5)	0.127 (6)	0.040 (3)	0.044 (4)	0.047 (5)
C27	0.0352 (7)	0.0334 (7)	0.0361 (7)	0.0027 (6)	0.0081 (6)	0.0141 (6)
C28	0.0403 (8)	0.0376 (8)	0.0431 (8)	0.0057 (6)	0.0047 (7)	0.0119 (7)
O1	0.0377 (5)	0.0324 (5)	0.0344 (5)	0.0049 (4)	0.0045 (4)	0.0106 (4)
O2	0.0534 (7)	0.0397 (6)	0.0768 (8)	0.0059 (5)	0.0227 (6)	0.0256 (6)
O3	0.0405 (6)	0.0424 (6)	0.0644 (7)	0.0132 (5)	0.0161 (5)	0.0219 (5)
O4	0.0597 (7)	0.0728 (8)	0.0491 (7)	0.0125 (6)	0.0080 (6)	0.0337 (6)
O5	0.0323 (9)	0.0749 (13)	0.0728 (16)	−0.0031 (8)	0.0011 (10)	0.0415 (13)
C29	0.0372 (14)	0.107 (2)	0.089 (2)	−0.0006 (13)	−0.0061 (14)	0.0509 (19)
C30	0.075 (3)	0.137 (3)	0.084 (3)	−0.027 (2)	−0.0103 (18)	0.053 (2)
O5'	0.037 (4)	0.087 (4)	0.073 (5)	−0.016 (3)	−0.001 (4)	0.046 (4)
C29'	0.037 (5)	0.110 (6)	0.079 (6)	−0.010 (4)	−0.006 (4)	0.042 (5)
C30'	0.063 (8)	0.126 (9)	0.110 (9)	−0.004 (7)	−0.010 (7)	0.048 (8)

### Geometric parameters (Å, º)

**Table:**

C1—O1	1.4626 (16)	C20—H20C	0.9600
C1—C16	1.4973 (19)	C21—C22	1.380 (2)
C1—C2	1.5161 (19)	C21—H21	0.9300
C1—C23	1.5528 (18)	C22—H22	0.9300
C2—C3	1.375 (2)	C23—C24	1.5039 (19)
C2—C7	1.383 (2)	C23—C27	1.5557 (19)
C3—C4	1.386 (2)	C23—H23	0.9800
C3—H3	0.9300	C24—O2	1.1918 (17)
C4—C5	1.375 (3)	C24—O3	1.3317 (17)
C4—H4	0.9300	C25—O3	1.4513 (19)
C5—C6	1.384 (2)	C25—C26'	1.464 (6)
C5—H5	0.9300	C25—C26	1.474 (8)
C6—C7	1.380 (2)	C25—H25A	0.9700
C6—H6	0.9300	C25—H25B	0.9700
C7—C8	1.518 (2)	C25—H25C	0.9700
C8—O1	1.4429 (16)	C25—H25D	0.9700
C8—C9	1.4967 (19)	C26—H26A	0.9600
C8—C27	1.5750 (19)	C26—H26B	0.9600
C9—C15	1.381 (2)	C26—H26C	0.9600
C9—C10	1.385 (2)	C26'—H26D	0.9600
C10—C11	1.384 (2)	C26'—H26E	0.9600
C10—H10	0.9300	C26'—H26F	0.9600
C11—C12	1.378 (2)	C27—C28	1.5082 (19)
C11—H11	0.9300	C27—H27	0.9800
C12—C14	1.385 (2)	C28—O5'	1.185 (12)
C12—C13	1.505 (2)	C28—O4	1.1908 (18)
C13—H13A	0.9600	C28—O5	1.367 (3)
C13—H13B	0.9600	O5—C29	1.448 (3)
C13—H13C	0.9600	C29—C30	1.506 (5)
C14—C15	1.382 (2)	C29—H29A	0.9700
C14—H14	0.9300	C29—H29B	0.9700
C15—H15	0.9300	C30—H30A	0.9600
C16—C22	1.379 (2)	C30—H30B	0.9600
C16—C17	1.386 (2)	C30—H30C	0.9600
C17—C18	1.375 (2)	O5'—C29'	1.464 (9)
C17—H17	0.9300	C29'—C30'	1.505 (10)
C18—C19	1.382 (3)	C29'—H29C	0.9700
C18—H18	0.9300	C29'—H29D	0.9700
C19—C21	1.374 (2)	C30'—H30D	0.9600
C19—C20	1.511 (2)	C30'—H30E	0.9600
C20—H20A	0.9600	C30'—H30F	0.9600
C20—H20B	0.9600		
			
O1—C1—C16	110.12 (10)	C22—C21—H21	119.1
O1—C1—C2	100.29 (10)	C16—C22—C21	120.49 (14)
C16—C1—C2	117.12 (11)	C16—C22—H22	119.8
O1—C1—C23	99.13 (10)	C21—C22—H22	119.8
C16—C1—C23	118.59 (11)	C24—C23—C1	116.13 (11)
C2—C1—C23	108.50 (11)	C24—C23—C27	116.58 (11)
C3—C2—C7	121.60 (13)	C1—C23—C27	102.32 (10)
C3—C2—C1	132.64 (13)	C24—C23—H23	107.1
C7—C2—C1	105.76 (12)	C1—C23—H23	107.1
C2—C3—C4	117.65 (15)	C27—C23—H23	107.1
C2—C3—H3	121.2	O2—C24—O3	124.22 (13)
C4—C3—H3	121.2	O2—C24—C23	126.09 (13)
C5—C4—C3	120.99 (15)	O3—C24—C23	109.61 (12)
C5—C4—H4	119.5	O3—C25—C26'	108.0 (4)
C3—C4—H4	119.5	O3—C25—C26	107.3 (6)
C4—C5—C6	121.24 (15)	C26'—C25—C26	26.7 (5)
C4—C5—H5	119.4	O3—C25—H25A	110.2
C6—C5—H5	119.4	C26'—C25—H25A	130.6
C7—C6—C5	117.93 (15)	C26—C25—H25A	110.2
C7—C6—H6	121.0	O3—C25—H25B	110.2
C5—C6—H6	121.0	C26'—C25—H25B	85.8
C6—C7—C2	120.57 (14)	C26—C25—H25B	110.2
C6—C7—C8	134.20 (14)	H25A—C25—H25B	108.5
C2—C7—C8	105.22 (12)	O3—C25—H25C	110.1
O1—C8—C9	112.25 (11)	C26'—C25—H25C	110.1
O1—C8—C7	100.69 (10)	C26—C25—H25C	86.1
C9—C8—C7	118.93 (12)	H25A—C25—H25C	26.2
O1—C8—C27	98.67 (10)	H25B—C25—H25C	128.7
C9—C8—C27	113.99 (11)	O3—C25—H25D	110.1
C7—C8—C27	109.66 (11)	C26'—C25—H25D	110.2
C15—C9—C10	118.17 (14)	C26—C25—H25D	131.3
C15—C9—C8	120.12 (13)	H25A—C25—H25D	84.6
C10—C9—C8	121.66 (13)	H25B—C25—H25D	26.4
C11—C10—C9	120.54 (15)	H25C—C25—H25D	108.4
C11—C10—H10	119.7	C25—C26—H26A	109.5
C9—C10—H10	119.7	C25—C26—H26B	109.5
C12—C11—C10	121.65 (15)	C25—C26—H26C	109.5
C12—C11—H11	119.2	C25—C26'—H26D	109.5
C10—C11—H11	119.2	C25—C26'—H26E	109.5
C11—C12—C14	117.43 (15)	C25—C26'—H26F	109.5
C11—C12—C13	121.41 (17)	C28—C27—C23	115.71 (11)
C14—C12—C13	121.17 (17)	C28—C27—C8	116.03 (11)
C12—C13—H13A	109.5	C23—C27—C8	101.22 (10)
C12—C13—H13B	109.5	C28—C27—H27	107.8
H13A—C13—H13B	109.5	C23—C27—H27	107.8
C12—C13—H13C	109.5	C8—C27—H27	107.8
H13A—C13—H13C	109.5	O5'—C28—O4	110.8 (6)
H13B—C13—H13C	109.5	O5'—C28—O5	26.3 (6)
C15—C14—C12	121.43 (16)	O4—C28—O5	126.40 (18)
C15—C14—H14	119.3	O5'—C28—C27	120.1 (6)
C12—C14—H14	119.3	O4—C28—C27	125.79 (14)
C9—C15—C14	120.77 (16)	O5—C28—C27	107.73 (16)
C9—C15—H15	119.6	C8—O1—C1	98.05 (9)
C14—C15—H15	119.6	C24—O3—C25	116.27 (12)
C22—C16—C17	118.01 (14)	C28—O5—C29	116.9 (2)
C22—C16—C1	123.37 (13)	O5—C29—C30	110.0 (3)
C17—C16—C1	118.62 (13)	O5—C29—H29A	109.7
C18—C17—C16	120.91 (15)	C30—C29—H29A	109.7
C18—C17—H17	119.5	O5—C29—H29B	109.7
C16—C17—H17	119.5	C30—C29—H29B	109.7
C17—C18—C19	121.23 (16)	H29A—C29—H29B	108.2
C17—C18—H18	119.4	C28—O5'—C29'	119.8 (10)
C19—C18—H18	119.4	O5'—C29'—C30'	104.5 (11)
C21—C19—C18	117.54 (15)	O5'—C29'—H29C	110.8
C21—C19—C20	121.38 (17)	C30'—C29'—H29C	110.8
C18—C19—C20	121.07 (17)	O5'—C29'—H29D	110.8
C19—C20—H20A	109.5	C30'—C29'—H29D	110.8
C19—C20—H20B	109.5	H29C—C29'—H29D	108.9
H20A—C20—H20B	109.5	C29'—C30'—H30D	109.5
C19—C20—H20C	109.5	C29'—C30'—H30E	109.5
H20A—C20—H20C	109.5	H30D—C30'—H30E	109.5
H20B—C20—H20C	109.5	C29'—C30'—H30F	109.5
C19—C21—C22	121.80 (15)	H30D—C30'—H30F	109.5
C19—C21—H21	119.1	H30E—C30'—H30F	109.5
			
O1—C1—C2—C3	−148.50 (15)	C18—C19—C21—C22	−0.7 (3)
C16—C1—C2—C3	−29.4 (2)	C20—C19—C21—C22	178.76 (18)
C23—C1—C2—C3	108.15 (17)	C17—C16—C22—C21	0.4 (2)
O1—C1—C2—C7	31.14 (13)	C1—C16—C22—C21	−179.34 (14)
C16—C1—C2—C7	150.21 (12)	C19—C21—C22—C16	−0.2 (3)
C23—C1—C2—C7	−72.21 (13)	O1—C1—C23—C24	−162.37 (11)
C7—C2—C3—C4	−1.6 (2)	C16—C1—C23—C24	78.66 (15)
C1—C2—C3—C4	177.97 (15)	C2—C1—C23—C24	−58.21 (15)
C2—C3—C4—C5	0.5 (2)	O1—C1—C23—C27	−34.26 (12)
C3—C4—C5—C6	0.9 (3)	C16—C1—C23—C27	−153.23 (11)
C4—C5—C6—C7	−1.2 (2)	C2—C1—C23—C27	69.90 (12)
C5—C6—C7—C2	0.1 (2)	C1—C23—C24—O2	−0.9 (2)
C5—C6—C7—C8	−178.61 (15)	C27—C23—C24—O2	−121.67 (16)
C3—C2—C7—C6	1.3 (2)	C1—C23—C24—O3	−177.87 (11)
C1—C2—C7—C6	−178.37 (13)	C27—C23—C24—O3	61.39 (15)
C3—C2—C7—C8	−179.61 (13)	C24—C23—C27—C28	0.28 (17)
C1—C2—C7—C8	0.70 (14)	C1—C23—C27—C28	−127.54 (12)
C6—C7—C8—O1	146.03 (16)	C24—C23—C27—C8	126.58 (12)
C2—C7—C8—O1	−32.84 (13)	C1—C23—C27—C8	−1.25 (12)
C6—C7—C8—C9	23.0 (2)	O1—C8—C27—C28	162.98 (11)
C2—C7—C8—C9	−155.84 (12)	C9—C8—C27—C28	−77.88 (15)
C6—C7—C8—C27	−110.65 (17)	C7—C8—C27—C28	58.28 (15)
C2—C7—C8—C27	70.48 (13)	O1—C8—C27—C23	36.89 (11)
O1—C8—C9—C15	−178.75 (13)	C9—C8—C27—C23	156.03 (11)
C7—C8—C9—C15	−61.68 (19)	C7—C8—C27—C23	−67.81 (13)
C27—C8—C9—C15	70.13 (18)	C23—C27—C28—O5'	−145.8 (9)
O1—C8—C9—C10	3.92 (18)	C8—C27—C28—O5'	95.8 (9)
C7—C8—C9—C10	120.99 (15)	C23—C27—C28—O4	56.7 (2)
C27—C8—C9—C10	−107.20 (15)	C8—C27—C28—O4	−61.7 (2)
C15—C9—C10—C11	−0.7 (2)	C23—C27—C28—O5	−120.24 (18)
C8—C9—C10—C11	176.67 (13)	C8—C27—C28—O5	121.37 (18)
C9—C10—C11—C12	0.4 (2)	C9—C8—O1—C1	178.96 (11)
C10—C11—C12—C14	0.6 (2)	C7—C8—O1—C1	51.43 (11)
C10—C11—C12—C13	−179.54 (17)	C27—C8—O1—C1	−60.60 (11)
C11—C12—C14—C15	−1.3 (3)	C16—C1—O1—C8	−174.89 (10)
C13—C12—C14—C15	178.88 (18)	C2—C1—O1—C8	−50.84 (11)
C10—C9—C15—C14	0.1 (2)	C23—C1—O1—C8	60.00 (11)
C8—C9—C15—C14	−177.36 (15)	O2—C24—O3—C25	4.4 (2)
C12—C14—C15—C9	0.9 (3)	C23—C24—O3—C25	−178.61 (13)
O1—C1—C16—C22	−122.79 (14)	C26'—C25—O3—C24	−160.0 (6)
C2—C1—C16—C22	123.55 (15)	C26—C25—O3—C24	172.0 (9)
C23—C1—C16—C22	−9.7 (2)	O5'—C28—O5—C29	−60.0 (15)
O1—C1—C16—C17	57.42 (17)	O4—C28—O5—C29	0.5 (4)
C2—C1—C16—C17	−56.24 (18)	C27—C28—O5—C29	177.4 (2)
C23—C1—C16—C17	170.51 (13)	C28—O5—C29—C30	89.0 (5)
C22—C16—C17—C18	0.2 (3)	O4—C28—O5'—C29'	−20.1 (14)
C1—C16—C17—C18	180.00 (16)	O5—C28—O5'—C29'	111 (2)
C16—C17—C18—C19	−1.1 (3)	C27—C28—O5'—C29'	179.3 (9)
C17—C18—C19—C21	1.3 (3)	C28—O5'—C29'—C30'	−176 (2)
C17—C18—C19—C20	−178.1 (2)		

### Hydrogen-bond geometry (Å, º)

**Table:**

*D*—H···*A*	*D*—H	H···*A*	*D*···*A*	*D*—H···*A*
C5—H5···O4^i^	0.93	2.66	3.558 (2)	164
C11—H11···O2^ii^	0.93	2.66	3.433 (2)	141

Symmetry codes: (i) −*x*, −*y*+2, −*z*; (ii) *x*, *y*−1, *z*.
